# Mapping Brain Response to Pain in Fibromyalgia Patients Using Temporal Analysis of fMRI

**DOI:** 10.1371/journal.pone.0005224

**Published:** 2009-04-21

**Authors:** Jesus Pujol, Marina López-Solà, Héctor Ortiz, Joan Carles Vilanova, Ben J. Harrison, Murat Yücel, Carles Soriano-Mas, Narcís Cardoner, Joan Deus

**Affiliations:** 1 Institut d'Alta Tecnologia-PRBB, CRC Corporació Sanitària, Barcelona, Spain; 2 Networking Research Center on Bioengineering, Biomaterials and Nanomedicine (CIBER-BBN), Barcelona, Spain; 3 Clinical Sciences Departament, Faculty of Medicine, University of Barcelona, Barcelona, Spain; 4 Department of Electronic Engineering, Technical University of Catalonia, Barcelona, Spain; 5 Ressonància Magnètica, Clínica Girona, Girona, Spain; 6 Melbourne Neuropsychiatry Centre, Department of Psychiatry, The University of Melbourne, Melbourne, Australia; 7 Department of Psychiatry, Bellvitge University Hospital, Barcelona, Spain; 8 Department of Clinical and Health Psychology, Autonomous University of Barcelona, Barcelona, Spain; University of Granada, Spain

## Abstract

**Background:**

Nociceptive stimuli may evoke brain responses longer than the stimulus duration often partially detected by conventional neuroimaging. Fibromyalgia patients typically complain of severe pain from gentle stimuli. We aimed to characterize brain response to painful pressure in fibromyalgia patients by generating activation maps adjusted for the duration of brain responses.

**Methodology/Principal Findings:**

Twenty-seven women (mean age: 47.8 years) were assessed with fMRI. The sample included nine fibromyalgia patients and nine healthy subjects who received 4 kg/cm^2^ of pressure on the thumb. Nine additional control subjects received 6.8 kg/cm^2^ to match the patients for the severity of perceived pain. Independent Component Analysis characterized the temporal dynamics of the actual brain response to pressure. Statistical parametric maps were estimated using the obtained time courses. Brain response to pressure (18 seconds) consistently exceeded the stimulus application (9 seconds) in somatosensory regions in all groups. fMRI maps following such temporal dynamics showed a complete pain network response (sensory-motor cortices, operculo-insula, cingulate cortex, and basal ganglia) to 4 kg/cm^2^ of pressure in fibromyalgia patients. In healthy subjects, response to this low intensity pressure involved mainly somatosensory cortices. When matched for perceived pain (6.8 kg/cm^2^), control subjects showed also comprehensive activation of pain-related regions, but fibromyalgia patients showed significantly larger activation in the anterior insula-basal ganglia complex and the cingulate cortex.

**Conclusions/Significance:**

The results suggest that data-driven fMRI assessments may complement conventional neuroimaging for characterizing pain responses and that enhancement of brain activation in fibromyalgia patients may be particularly relevant in emotion-related regions.

## Introduction

Nociceptive stimulation can trigger complex behavioral responses involving both local pain sensations and general affective phenomena [1]. Responses to painful mechanical stimuli typically persist after their application for a time that largely depends on stimulus features and the individual's receptive state [2], [3].

Functional imaging has notably contributed to delineating the functional anatomy of the brain network mediating pain responses [4]. The most consistent activations in this “pain matrix” involve somatosensory and adjacent parietal cortex, the operculo-insular region and the anterior cingulate cortex [see specific reviews 1], [4], [5]. Interestingly, only a few imaging studies have explored nociception temporal dynamics, suggesting that pain-related activity may persist well beyond the specified stimulation periods [3], [6]–[10].

Fibromyalgia is a syndrome expressed mainly as chronic complaints involving augmented subjective pain of mechanical origin [11]. Previous functional magnetic resonance imaging (fMRI) studies assessing the anatomy of brain activations have suggested that brain responses to mechanical stimuli are abnormally increased in fibromyalgia patients [12]. In this study, we aimed to further characterize brain response to pain in patients with severe fibromyalgia and healthy subjects using an fMRI data-driven approach [13], [14]. We assessed the temporal dynamics of the actual brain response to local painful pressure in pain-related regions with Independent Component Analysis (ICA). The results were then used to generate fMRI maps adjusted for the duration of brain responses that showed more complete activation patterns in patients and in control subjects and stronger correlation with reported subjective pain.

## Methods

### Ethics statement

This study was conducted according to the principles expressed in the Declaration of Helsinki. The study was approved by the Ethics and Institutional Review Board of the Autonomous University of Barcelona (reference number SAF2007-62376). All patients and healthy subjects provided written informed consent for clinical and fMRI assessment and subsequent analyses.

### Subjects

Twenty-seven subjects participated in the study, including nine patients with fibromyalgia and two groups of nine healthy subjects (control group 1 and 2) matched to patients for gender and age, and recruited from the same sociodemographic environment. Control group 1 served to compare brain response to a fixed mechanical stimulus pressure able to provoke severe pain in fibromyalgia patients. Control group 2 was matched to fibromyalgia patients for levels of perceived pain by increasing stimulus intensity.

The patients were consecutively selected during clinical follow-up to make up a homogeneous sample showing severe and durable symptoms. The series included nine right-handed females with a mean±SD age of 47.9±9.4 years and education level of 11.0±2.1 years. All patients met the American College of Rheumatology criteria for fibromyalgia [11]. Mean illness duration was 8.2±5.6 years. The number of tender points upon study assessment was 16.7±2.3. General Perception of Health according to the 36-Item Short-Form Health Survey [15] scored 11.1±13.2 (maximum score, 100). The Fibromyalgia Impact Questionnaire (FIQ) [16] total score was 73.2±13.8 (maximum score, 100). Hospital Anxiety and Depression Scale (HADS) ratings [17], [18] were 13.4±4.0 and 10.3±4.7. One patient had a co-morbid clinical diagnosis of major depression, 2 patients a dysthymic disorder and 3 patients an adjustment disorder with mixed anxiety and depressed mood.

Patients were allowed to continue with their stable medical treatment, but were required to refrain from taking analgesic drugs 72 hours prior to fMRI. Six patients were on anti-inflammatory drugs in a stable regime (2 were also taking benzodiazepines, 1 antidepressants and 1 carbamazepine). The remaining 3 patients were taking: antidepressants, benzodiazepines and carbamazepine (1 patient), antidepressants and benzodiazepines (1 patient), and no medication (1 patient).

The control group 1 included nine right-handed females with a mean age of 47.2±8.9 years and education level 12.4±4.3 years, and the control group 2 nine right-handed females with a mean age of 48.2±5.5 years and education level 13.0±3.0 years. Subjects with relevant medical or neurological disorder, substance abuse, or psychiatric disease were not considered for inclusion. None of the healthy subjects was undergoing medical treatment.

### Stimuli

Pressure stimuli were delivered using a specially designed hydraulic device capable of transmitting controlled pressure to 1-cm^2^ surface placed on the subject's thumbnail. As in other studies [19], [20], this system involved a hard rubber probe attached to a hydraulic piston that was displaced by mechanical pressure. In a preliminary session, each subject was acclimatized to the mechanical stimuli and trained to rate perceived pain intensity using a numerical rating scale (NRS) ranging from 0 (no pain) to 100 (the worst pain possible).

Pain thresholds were also assessed during the session and the intensity of pressure producing severe pain in both patients and control subjects was estimated. To determine individual thresholds, different stimulus intensities were applied lasting 5 seconds each, with an inter-stimuli interval of 20 seconds. The selected pressure stimuli, ranging from 2–9 kg/cm^2^, were administered pseudo-randomly. Conventional pain thresholds corresponded to the least pressure intensity at which subjects perceived pain in two trials. In this session, pain threshold was 1.6±0.5 kg/cm^2^ in the 9 patients and 4.0±1.0 kg/cm^2^ in the 18 healthy subjects (P<0.0005). The minimum pressure intensity to provoke severe pain (NRS above 70) in patients was 3.6±0.9 kg/cm^2^ and 6.8±1.4 kg/cm^2^ in healthy subjects (P<0.0005).

### fMRI pain paradigm

During the primary study assessment, identical stimulation was applied to both patients and healthy subjects (control group 1). A block-design paradigm was used consisting of 21-second resting-state periods interleaved with pressure stimulation blocks of nine seconds. During pressure blocks, sustained 4 kg/cm^2^ pressure was delivered to the subjects' right thumbnail. Pressure was partially removed for 1 second in the middle of each pain block to reduce the probability of tissue damage in the thumb. The entire imaging sequence involved 12 rest-pressure cycles lasting 6 minutes in total. Immediately after image acquisition, each subject provided a single score to globally rate pain intensity perceived during the 12 pressure blocks.

The control group 2 was assessed using identical procedures, but applying 6.8 kg/cm^2^, which produced a pain severity level similar to that experienced by fibromyalgia patients using 4 kg/cm^2^ (NRS above 70).

### MRI acquisition

A 1.5 T Signa system (General Electric, Milwaukee, WI) equipped with an eight-channel phased-array head coil and single-shot echoplanar imaging (EPI) software was used. Functional sequences consisted of gradient recalled acquisition in the steady-state (time of repetition [TR], 3,000 ms; time of echo [TE], 50 ms; pulse angle, 90°) within a field of view of 24 cm, a 96×64-pixel matrix, and slice thickness of 5 mm (inter-slice gap, 1 mm). Seventeen slices parallel to the anterior-posterior commissure line covered the whole-brain. The first two images in each run were discarded to allow the magnetization to reach equilibrium.

### Image preprocessing

Imaging data were processed using MATLAB version 7 (The MathWorks Inc, Natick, Mass) and Statistical Parametric Mapping software (SPM5; The Wellcome Department of Imaging Neuroscience, London). Preprocessing involved motion correction, spatial normalization and smoothing using a Gaussian filter (full-width half-maximum, 6 mm). Data were normalized to the standard SPM-EPI template and resliced to 3 mm isotropic resolution in Montreal Neurological Institute (MNI) space. We excluded data from two subjects from an original sample of 29 subjects due to excessive head movement (z-axis translation>2 mm).

### Image analysis

fMRI data are commonly analyzed using ‘model-based’ statistical methods that require a specific assumption about the time courses of activation. Typically, model-based analyses estimate the contrast between signal intensity of images obtained during stimulus application and signal intensity of images obtained without stimulation or during a control condition. In experiments where response durations cannot be completely anticipated, as in pain assessment and in the assessment of emotions in general, the standard model-based approach may underestimate the evoked brain response. In contrast, “data-driven” statistical methods are used to identify actual brain activation without a priori hypothesis on the expected activation time course. These methods estimate the best fitting of the data, but do not directly test the statistical significance of the activations [13], [14]. In the current study, we used a data-driven approach based on Independent Component Analysis (ICA) to generate a study-specific time course model, which was used as a regressor in conventional SPM analyses to statistically test between-group differences for the activation pattern.

### Independent Component Analysis

Spatial Independent Component Analysis is a data-driven statistical analysis method that is able to decompose whole brain fMRI data into independent networks of brain regions (spatial components) involving voxels following similar temporal dynamics. Results are presented as a set of spatial maps with their associated time courses.

Group ICA for fMRI Toolbox was used (GIFT v1.3c; http://icatb.sourceforge.net), with previously described algorithms [21], [22]. After subject-wise data concatenations, a separate spatial ICA was performed for each study group in three stages: *Stage 1:* The dimensionality of the fMRI data and the optimal number of components for each group were estimated using the minimum description length (MDL) criterion in GIFT [23]. Principal component analysis (2 reduction steps) was then used to reduce individual subject data in dimensionality (for computational feasibility) to the number of components estimated by the MDL criterion. *Stage 2:* Group estimation of spatially independent sources was then performed using the Infomax algorithm. *Stage 3:* During the final stage of back-reconstruction to the original dimensionality, individual subject image maps and time courses were estimated using the group solution [21], [22]. This step was followed by the process of grouping components across subjects to produce group component maps and group-average time courses.

### Temporal analysis of brain response to pain

Group ICA results were used to identify actual response functions (i.e., normalized time courses) of the brain regions activated by nociceptive stimulation. In selecting these time courses for further analysis, we considered those components involving regions known to mediate brain response to pain [4] and showing a consistent signal increase (activation) coinciding with each pain stimulation block, irrespectively of the duration of the activation.

### Mapping brain response to pain: analyses of main task effects

1st-level (single-subject) SPM contrast images were estimated to characterize the functional anatomy of pain-related brain activations. For this analysis, the BOLD response at each voxel was modeled using (i) data-driven response function generated from the Group ICA; and (ii) conventional (SPM5) model-driven canonical hemodynamic response function. Resulting 1st-level contrast images for each subject were then carried forward to 2nd-level random-effects (group) analyses using one-sample t-tests. A two-sample t-test analysis was performed to compare activation maps between study groups. Spatial coordinates from the obtained maps were then converted to standard Talairach coordinates [24] using a non-linear transform of SPM standard space to Talairach space [25].

### Mapping brain response to pain: correlation maps

We mapped voxel-wise correlations between subjective pain scores and brain activation. Separate correlation maps were obtained for both the data-driven and model-driven approaches including 18 study subjects (patients and control group 1). Correlations were considered significant at a *P* value less than 0.05 False Discovery Rate (FDR) corrected for the volume of activated regions (pain network).

In addition, we assessed the extent to which brain activation in the region showing the highest correlation with subjective pain (the anterior cingulate cortex) was able to account for group differences in perceived pain. This was carried out by comparing group differences in subjective reported pain both before and after controlling for (regressing out) the effect of cingulate activation using analysis of covariance (ANCOVA).

## Results

### Pain rating during fMRI assessment

The range of subjectively reported pain varied from 20 to 100 points across the 18 subjects (9 patients and 9 healthy subjects from the control group 1) assessed using 4 kg/cm^2^ of pressure. Healthy subjects reported mild-to-moderate pain and fibromyalgia patients the most severe scores during this stimulation (mean±SD for healthy subjects = 41.1±20.1 and for patients 88.8±11.6; *t* = 6.2 and *P*<0.0001). The group of healthy subjects (n = 9) receiving 6.8 kg/cm^2^ (control group 2) reported severe pain at rating levels comparable to the fibromyalgia group (80.2±10.7; *t* = 1.6 and *P* = 0.123).

### Temporal analysis of brain activation at 4 kg/cm^2^ of pressure

ICA estimated 34 spatially independent components in patients and 31 in healthy subjects (control group 1). The time course of nine components in patients and three components in healthy subjects showed a signal increase (i.e., activation) coinciding with each pain stimulation block. Two such components involved pain-related brain regions in each study group. That is, in both patients and healthy subjects, a “somatosensory” and an “insular” component met the double criterion of showing signal increase in each pain block and involving regions known to mediate brain response to pain.

**Figure 1:**
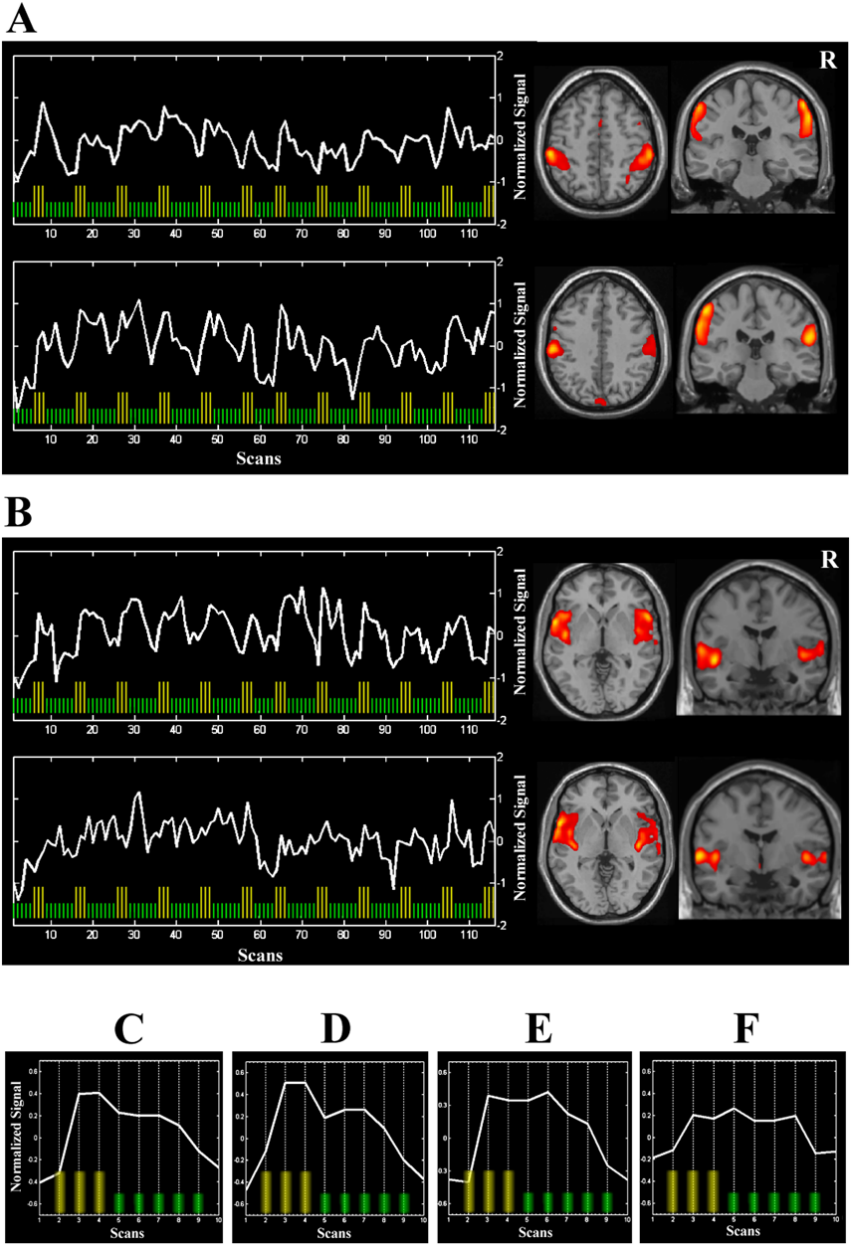
Temporal dynamics of the brain response to painful stimulation. (A) shows time courses and representative brain slices for the somatosensory component in fibromyalgia patients (top) and healthy subjects (bottom) derived from activation temporal analysis. (B) shows the corresponding data for the insular component in patients (top) and healthy subjects (bottom). (C–F) show block-average time courses for the somatosensory component in patients (C) and healthy subjects (D), and the insular component in patients (E) and healthy subjects (F). Yellow bars identify stimulation scans. R indicates right hemisphere.

The somatosensory component included bilateral parietal cortex in both groups and a small portion of the dorsal anterior cingulate cortex in fibromyalgia (Figure 1). The associated time course was very similar in patients and healthy subjects showing evoked signal changes that persisted after stimulus removal in each stimulation block. Block-average time courses (Figure 1) revealed an early fMRI signal increase that returned to the baseline level only after 18 seconds in both groups (twice the duration of the applied stimulus). Time to peak activation since stimulus onset was 6.9±5.1 s in patients and 6.3±4.8 s in control subjects (control group 1), showing *t* = 0.28 and *P* = 0.782. Activation duration was 18.6±3.6 s in patients and 18.9±3.6 s in control subjects, showing *t* = −0.19 and *P* = 0.848.

The “insular” component involved bilateral insulo-opercular cortex in both groups. In fibromyalgia patients, the time course of this component followed the dynamics of the somatosensory component, showing a fast initial signal increase and duration of 18 seconds. By contrast, healthy subjects, showed much less consistent signal changes in the insular region, as not all the stimulation blocks showed a definite signal increase (see Figure 1B).

### Mapping brain response to 4 kg/cm^2^ of pressure

**Figure 2:**
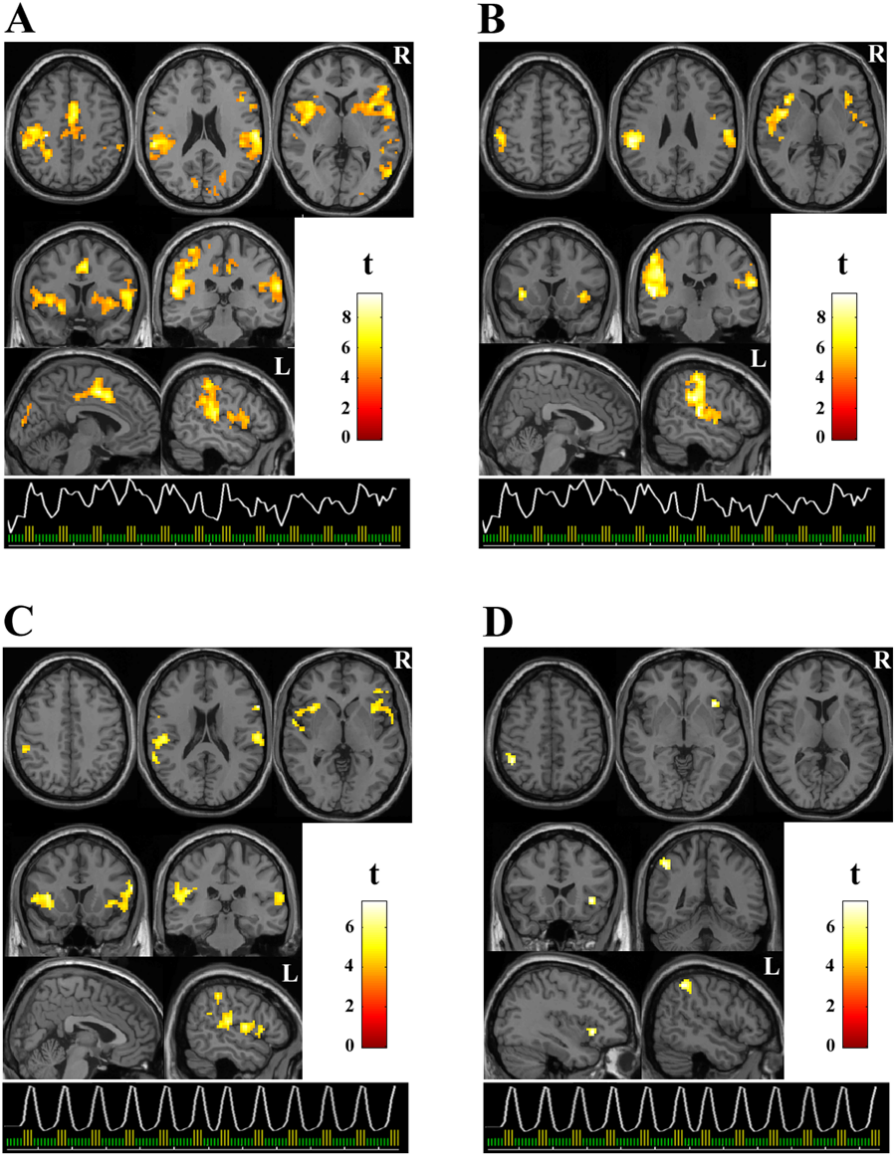
Brain activation maps. Brain response to 4 kg/cm^2^ of pressure applied on the right thumb. Statistical parametric maps (SPM) are shown adjusted for response duration in fibromyalgia patients (A) and healthy subjects (B), and for stimulus duration in patients (C) and healthy subjects (D). Graphs illustrate the reference function models used in the SPM analysis (i. e., the time course from the somatosensory component averaged across groups in both A an B, and conventional canonical hemodynamic response function in C and D). Display threshold, *P*<0.0005, 20 voxels for all the data. R and L indicate right and left hemispheres. The names of the regions are shown in Table 1 and 2.

The time course of the somatosensory component was averaged across groups (patients and control group 1) and was used as the reference function in a conventional fMRI analysis. Figure 2 and Table 1 report brain activations obtained using this data-driven model. In fibromyalgia patients, activations involved all relevant regions of the pain network, including contralateral somatosensory and motor cortices, bilateral inferior parietal areas, the opercula, the insula, the basal ganglia, the supplementary motor area, the anterior cingulate cortex and the cerebellum. In healthy controls, activation was mainly observed in the inferior parietal cortex involving the supramarginal gyrus, and in the insula. Statistical differences between both groups are reported in Table 1.

**Table 1:** Brain Activations Adjusted for Response Duration (Data-Driven Analysis).

	Fibromyalgia		Healthy Controls		Patients>Controls	
	*z*	*X∶Y∶Z*	*z*	*X∶Y∶Z*	*z*	*X∶Y∶Z*
**Sensory-Motor Cortex**	4.4	−36∶−17∶59	4.9	−51∶−27∶48	3.9	−45∶−15∶42
**Inferior Parietal - SII**	4.8	−48∶−28∶26	5.1	−54∶−28∶26	3.0	−45∶−36∶30
	4.0	59∶−37∶21	5.0	56∶−17∶17		
	5.4	−59∶−28∶18	5.0	−59∶−23∶15	3.7	−59∶−28∶18
	5.1	56∶−17∶17				
**Insula**	4.9	−39∶15∶5	4.8	−36∶15∶2	3.5	−45∶18∶7
	4.9	36∶15∶−1	4.3	36∶15∶−1		
**Anterior Cingulate - SMA**	5.1	0∶14∶38			3.9	0∶14∶38
	5.3	3∶2∶44			4.1	3∶2∶44
	4.4	0∶0∶53				
**Basal Ganglia**	4.5	−27∶6∶−3			2.8	−27∶6∶−3
	4.4	15∶14∶−6			3.5	15∶14∶−6
**Other Regions:**						
**Angular Gyrus**	4.5	54∶−64∶6				
**Visual Cortex**	4.0	21∶−78∶18				
**Frontal Operculum**					4.0	48∶29∶4

Group activations show *P*<0.05 False Discovery Rate (FDR) whole brain corrected. The contrast patients>controls shows *P*<0.05 FDR corrected for the volume of activated regions (pain network). Coordinates (mm) are in the standard Talairach space. SII, second somatosensory cortex, SMA, supplementary motor area.

The assessment of brain activations from the conventional block-design analysis adjusted to stimulus duration (i.e., model-based) resulted in notably smaller pain-related activation in patients and control group 1 (Figure 2, Table 2).

**Table 2:** Brain Activations Adjusted for Stimulus Duration (Model-Based Analysis).

	Fibromyalgia		Healthy Controls		Patients>Controls	
	*z*	*X∶Y∶Z*	*z*	*X∶Y∶Z*	*z*	*X∶Y∶Z*
**Sensory-Motor Cortex**	4.2	−54∶−33∶43	4.4	−51∶−44∶49	3.7	−27∶−39∶46
**Inferior Parietal - SII**	4.3	−45∶−25∶23			3.4	−56∶−46∶22
	4.3	59∶−17∶20				
**Insula**	4.5	−39∶18∶5			3.8	−45∶18∶7
	3.9	36∶15∶0	4.5	36∶20∶−4		
**Frontal Operculum**					3.4	51∶29∶4

Group activations show *P*<0.05 False Discovery Rate (FDR) whole brain corrected. The contrast patients>controls shows *P*<0.05 FDR corrected for the volume of activated regions (pain network). Coordinates (mm) are in the standard Talairach space. SII, second somatosensory cortex.

### Correlation maps

**Figure 3:**
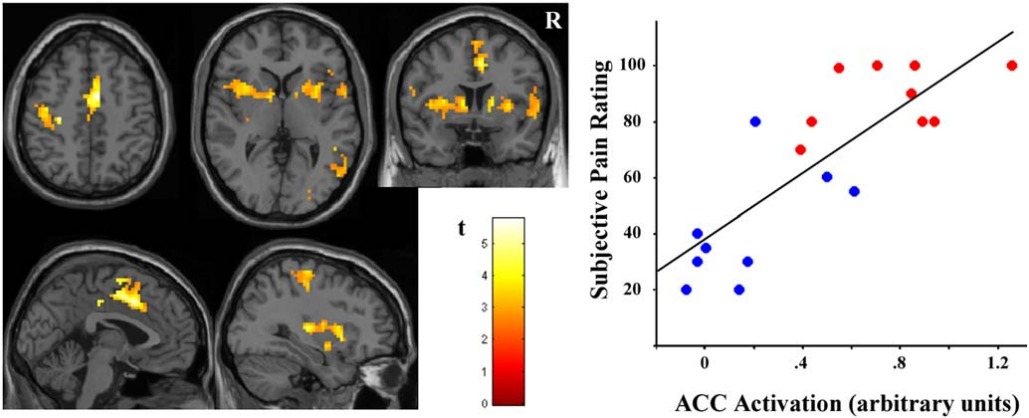
Correlation map between subjective pain scores and brain activations. (Adjusted for response duration -data-driven analysis- including all individuals). Display threshold, *P*<0.01, 10 voxels. R indicates right hemisphere. The plot illustrates the correlation at peak activation in anterior cingulate cortex (ACC) (*r* = 0.82, *P*<0.0001 and adjusted *r^2^* = 0.66). A.u., arbitrary units. Red and blue dots correspond to patients and control subjects, respectively. The names of the regions are shown in Table 3.

We mapped the correlation of subjective pain scores with brain activations during stimulation at 4 kg/cm^2^ of pressure (i.e., voxel-wise regression of the activation patterns with subjects' pain scores). Pain scores were widely correlated with brain activation in the data-driven approach involving the contralateral sensory-motor cortex, supplementary motor area, anterior cingulate cortex, anterior insula and basal ganglia (Figure 3, Table 3). By contrast, subjective pain showed no significant correlation with the activation pattern identified using the conventional model-driven approach.

**Table 3:** Correlation of Subjective Pain Scores with Brain Activations Adjusted for Response Duration (Data-Driven Analysis) (n = 18).

	Pearson	*z* score	Talairach Coor.
	*r*		*X∶Y∶Z*
**Sensory-Motor Cortex**	.73	3.4	−45∶−15∶42
**Inferior Parietal - SII**	.74	3.5	−59∶−28∶18
**Insula**	.69	3.2	−39∶15∶5
	.73	3.4	33∶12∶−1
**Anterior Cingulate – SMA**	.81	4.1	3∶11∶38
	.82	4.2	6∶2∶44
	.74	3.5	3∶0∶55
**Basal Ganglia**	.68	3.1	−33∶9∶5
	.63	2.8	18∶12∶−1
**Other Regions:**			
**Angular Gyrus**	.62	2.7	54∶−61∶3
**Frontal Operculum**	.64	2.9	56∶6∶3

All correlations show *P*<0.05 False Discovery Rate (FDR) corrected for the volume of activated regions (pain network).

The plot in Figure 3 shows a relatively graded correlation between subjective pain and anterior cingulate cortex activation when including all subjects stimulated at 4 kg/cm^2^ of pressure. Nevertheless, it is evident that patients and healthy subjects are at opposite extremes of the pain score range. Using ANCOVA, cingulate cortex activation was found to account largely for the differences between both groups in perceived pain. In this analysis, group differences in subjective pain scores were highly significant before controlling for the effect of anterior cingulate cortex activation (*F* = 38.0, *P*<0.0001); a finding that was reversed (*F* = 1.8, *P* = 0.195) when removing (regressing out) this effect.

### Comparing patients and controls subjects matched for pain levels

**Figure 4:**
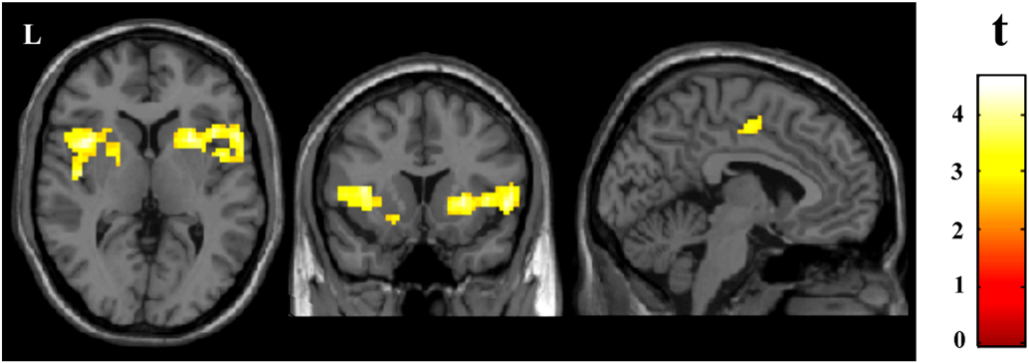
Comparison of fibromyalgia patients with healthy subjects matched for subjective pain levels. The statistical parametric map (SPM) adjusted for response duration shows the regions where patients receiving 4 kg/cm^2^ of pressure showed greater activation than control subjects receiving 6.8 kg/cm^2^. Display threshold, *P*<0.01, 10 voxels. L indicates left hemisphere. The names of the regions are shown in Table 4.

An ICA was carried out for the control group stimulated at pressure 6.8 kg/cm^2^ and reporting severe pain (control group 2). This procedure estimated 37 spatially independent components. As in the above analysis, the time course of the obtained somatosensory component was averaged with the somatosensory time course of fibromyalgia patients and was used as the reference function in a new conventional fMRI analysis to compare patients with this control group. Table 4 shows the activation pattern obtained in both groups and the significant between-group differences. Brain response was comprehensive both in patients and control subjects involving most of the pain-related regions. Response in regions involved in the sensory aspects of nociception was similar, showing a tendency for higher activation in the somatosensory cortex in control subjects. Patients, however, showed significantly greater activation in the anterior insula and basal ganglia bilaterally, and in the SMA (Table 4, Figure 4).

**Table 4:** Comparison analysis matching groups for subjective pain levels.

	Fibromyalgia (4 kg/cm^2^)		Healthy Controls (6.8 kg/cm^2^)		Patients>Controls	
	*z*	*X∶Y∶Z*	*z*	*X∶Y∶Z*	*z*	*X∶Y∶Z*
**Sensory-Motor Cortex**	4.6	−51∶−27∶43	5.8	−54∶−15∶48		
	4.0	54∶−15∶48	5.0	−33∶−29∶62		
			4.4	54∶−21∶48		
**Inferior Parietal - SII**	5.0	−48∶−20∶18	4.8	−60∶−22∶26		
	4.7	56∶−16∶23	4.9	56∶−16∶23		
**Insula**	5.0	−33∶−3∶8	4.8	−48∶−20∶18	3.6	−42∶12∶5
	4.6	−45∶−8∶6	4.4	−33∶−2∶11	3.6	38∶18∶−1
	4.7	39∶17∶−1	4.2	39∶−3∶−2		
**Anterior Cingulate - SMA**	4.8	0∶−1∶44	4.9	−6∶−1∶36	3.0	0∶−4∶44
	4.5	0∶0∶55	4.0	0∶0∶55		
**Basal Ganglia**	4.3	−27∶3∶−3			2.7	−27∶3∶8
	4.3	15∶12∶−1			3.6	30∶12∶−3
**Other Regions:**						
**Frontal Lobe**	3.7	56∶10∶16	4.0	54∶13∶35		
**Left Cerebellum**			4.8	−30∶−62∶−17		

Group activations show *P*<0.05 False Discovery Rate (FDR) whole brain corrected. The contrast patients>controls show *P*<0.05 FDR corrected for the volume of activated regions (pain network). Coordinates (mm) are in the standard Talairach space. SII, second somatosensory cortex, SMA, supplementary motor area. No significant findings were obtained in the contrast controls>patients.

## Discussion

This study aimed to characterize brain response to local pressure stimulation in fibromyalgia patients using an fMRI approach based on the temporal analysis of brain activation. Somatosensory areas showed consistent activation to each block of pressure stimulation that characteristically persisted beyond stimulus application. The fMRI maps adjusted for response duration showed robust activations in regions known to mediate brain responses to pain. Importantly, a strong correlation was observed between the rating of subjective pain during the fMRI assessment and the magnitude of the activation. Fibromyalgia patients showed significantly greater activation than comparative control subjects. Response enhancement was observed in fibromyalgia patients for most pain-related regions compared to the control subjects receiving identical stimulation, and for specific regions when the groups were matched for subjective pain levels.

This data-driven imaging analysis allowed us to compare specific temporal and anatomical features of nociceptive processing between fibromyalgia patients, who reported severe subjective pain to the relatively mild local pressure stimulus, and healthy subjects reporting only mild-to-moderate pain from this stimulation. We observed a similar activation time course in somatosensory cortices in both groups, which suggested relevant and durable responses to mechanical stimulation at the “sensory” stage of nociceptive processing, irrespectively of subjective pain severity. For the insula component, consistent long-lasting responses were observed only in fibromyalgia patients.

The anatomy of the activations in response to 4 kg/cm^2^ of pressure differed between patients and control subjects (control group 1). Healthy subjects showed mainly a sensory response with relevant activation in contralateral somatosensory cortices and moderate activation in the insular cortex. By contrast, fibromyalgia patients showed a full response to pain with robust sensory, limbic and motor activations. Functional MRI changes in these regions showed a significant correlation with the severity of experienced pain and largely accounted for group differences in subjective pain scores at low pressure stimulation. That is, increased activation in pain-related regions explained the increased subjective pain ratings in fibromyalgia patients.

It is noteworthy that all the “efferent” elements of the pain response (brain regions directly related to motor or visceral output) are represented in the voxel-wise map of the correlation between pain severity and brain activations, including contralateral sensory-motor cortex, supplementary motor area, anterior cingulate cortex, anterior insula and basal ganglia. Several fMRI studies have reported a close relationship between anterior cingulate cortex activation and the subjective experience of pain or its “suffering” component [2], [4]. This has been an especially robust finding in fMRI pain studies [2], [4], [26]–[28] and our results further support such an association. In addition, the reported map suggests that the other elements of the efferent pain response may also participate in the subjective experience of pain. Staud et al. [10] have reported a near identical pattern by mapping the correlation of perceived pain and brain activation related to temporal summation of “second pain” (late c-fiber evoked responses) during painful heat stimulation. Nevertheless, we did not obtain specific measurement of affect or unpleasantness during fMRI (only pain intensity ratings were recorded), which is a major limitation of our study. It would be of interest in future studies to map the correlation of brain activation during painful stimulation and individual affect ratings in addition to the reported correlation with pain intensity.

This closer correlation of subjective pain with the efferent brain response seems to further support proposed mechanisms for enhancement of emotions, including pain. Such models suggest that efferent somatic and visceral bodily responses to emotive stimuli originate backward afferent stimulation of the body representation in the brain, in turn amplifying emotional states [29]–[31]. Interestingly, the map showing the correlation of perceived pain with brain activations in our study largely coincides with the neural network related to interoceptive awareness in recent fMRI studies, which is proposed to mediate subjective feeling states arising from brain representations of bodily responses [32], [33]. Our data indeed suggest that fibromyalgia patients show enhanced responses in regions related to the individuals' emotion expression that may be part of the subjective pain experience. Nevertheless, these activations are not necessarily the result of augmented responses in the basic levels of nociceptive processing. A very recent study by Burgmer et al. [34] showed that abnormal brain responses in emotion-related regions in patients with fibromyalgia may be delayed with respect to peripheral painful stimulation, suggesting that their painful experience enhancement is likely to originate from central factors related to the patients' affect and cognition. Our study is limited in that the influence of these factors (e.g., patients' anxiety and depression) was not controlled in the analysis.

Our results are consistent with most of previous fMRI studies on fibromyalgia, but expand the reported data by assessing the temporal dynamics of brain activity, which led to a more comprehensive activation mapping. All the reports coincide in showing abnormal brain responses to painful stimuli in fibromyalgia patients [20], [35], [36] when comparing patients to control subjects receiving identical stimulus intensity. In general, the data are consistent with a model of enhanced normal pain response and argues against the occurrence of “aberrant” nociception [20], [37]. However, when matching both groups for perceived pain we observed larger activations in patients for specific regions. In this matching comparison, Gracely et al. [20] did not report significant differences between patients and control subjects with stimulation producing moderate pain. More recently, Staud et al. [9] specifically assessed the temporal summation of second pain using heat stimulation and also found no brain activation differences when stimulus strength was adjusted to induce moderate pain in both groups. In contrast with these two studies, more intense stimulation was used in our assessment and both patients and this control group reported severe pain. Fibromyalgia patients showed greater activation in the insula, basal ganglia and the anterior cingulate cortex, which are part of the brain network mediating efferent aspects of the pain response, and not in somatosensory cortices, where control subjects even had a tendency to show larger activation. Overall, our findings may be consistent with the notion of augmented brain response to pain in fibromyalgia, but the functional alterations may be particularly relevant in emotion-related (paralimbic) regions.

Functional MRI research is now focused on assessing the different dimensions of nociceptive processing. The presence of mood depression in fibromyalgia patients was associated with increased activation in regions processing affective components of pain [38]. Pain “catastrophizing,” or characterizations of pain as awful, horrible and unbearable, was related to increased activation in the attentional, affective and motor domains, independently of the influence of depression [19]. Another study suggested that patients' beliefs about pain-control (locus of control for pain) may influence nociceptive processing at the sensory-discriminative stage [39]. In this context, mapping brain activations adjusted to the temporal dynamics of each nociception dimension in different clinical and experimental situations may be of interest to further characterize the complex phenomenology of pain responses. Interestingly, Burgmer et al. [34] suggested that patients with fibromyalgia may show different temporal dynamics in different elements of the brain pain network.

Conventional block-design fMRI is based on detecting brain activations following a specified paradigm of stimulus duration. These methods provide reliable and accurate activation patterns when stimulus duration corresponds well to brain activation (typical in most sensory and motor tasks). Nevertheless, for painful or emotional stimuli that may evoke responses of variable duration, the temporal analysis of brain activity may provide more informative activation maps and correlate better with subjective pain scores. Data-driven methods, however, are inherently biased to the actual response in a given population or experiment, which may hinder the generalization of conclusions [13], [14]. For example, between-group comparisons may be difficult when the data-driven analyses identify different time courses for each group. In our study, it was feasible to compare groups using a common temporal model, as both patients and controls showed similar time courses for the somatosensory component.

Despite the small number of subjects included in this study, we observed robust activation maps reflecting the consistency of brain activation across all 12 pressure stimulation blocks. This may have particular relevance in the clinical fMRI setting as discussed in recent studies [40] where obtaining consistent findings at the individual case level is most desirable. Nonetheless, further studies will be needed to extrapolate our findings to the general population of fibromyalgia patients. In this context, it is also of interest to better establish the possible confounding effects of relevant clinical variables as, for example, the medication history of patients. In our study, no analgesic drugs were permitted 72 hours prior to fMRI, but patients were allowed to continue with their stable medical treatment, involving drugs with potential ability to modify the central nociceptive processing. In our patients, however, it is unlikely that the observed response enhancement to painful stimuli was a consequence of ongoing medical treatments, as the available data suggest the opposite effect [41]–[44]. Indeed, psychotropic medication showed no significant changes or ameliorative effects on abnormal functional neuroimaging measurements [43], while antidepressants reduced limbic activation during emotional processing [41], benzodiazepines reduced brain activity associated with anticipation to pain [44], and non-steroidal anti-inflammatory drugs suppressed pain-induced activation in most regions involved in pain processing [42].

Fibromyalgia has often been a controversial medical syndrome since patient identification is based largely on subjective symptoms [45]. In this and other studies [12], fMRI has demonstrated increased brain responses in patients labeled with this clinical diagnosis. Future research will establish the clinical usefulness of imaging tools for the objective assessment of subjective symptoms in both this and related disorders.
